# Therapeutic Potential of Quercetin Loaded Nanoparticles: Novel Insights in Alleviating Colitis in an Experimental DSS Induced Colitis Model

**DOI:** 10.3390/biomedicines10071654

**Published:** 2022-07-09

**Authors:** Safaa I. Khater, Marwa M. Lotfy, Maher N. Alandiyjany, Leena S. Alqahtani, Asmaa W. Zaglool, Fayez Althobaiti, Tamer Ahmed Ismail, Mohamed Mohamed Soliman, Saydat Saad, Doaa Ibrahim

**Affiliations:** 1 Department of Biochemistry, Faculty of Veterinary Medicine, Zagazig University, Zagazig 44511, Egypt; safaa_khater83@yahoo.com (S.I.K.); ssabelmageed@vet.zu.edu (S.S.); 2 Faculty of Pharmacy, Zagazig University, Zagazig 44511, Egypt; marwam14@yahoo.com; 3 Laboratory Medicine Department, Faculty of Applied Medical Sciences, Umm Al-Qura University, Makkah 21955, Saudi Arabia; mnandiyjany@uqu.edu.sa; 4 Quality and Development Affair, Batterjee Medical College, Jeddah 21442, Saudi Arabia; 5 Department of Biochemistry, College of Science, University of Jeddah, Jeddah 80203, Saudi Arabia; lsalqahtani@uj.edu.sa; 6 Department of Animal Wealth Development, Genetic and Genetic Engineering, Faculty of Veterinary Medicine, Zagazig University, Zagazig 44511, Egypt; asmaa.wagih2008@gmail.com; 7 Department of Biotechnology, College of Science, Taif University, P.O. Box 11099, Taif 21944, Saudi Arabia; faiz@tu.edu.sa; 8 Department of Clinical Laboratory Sciences, Turabah University College, Taif University, P.O. Box 11099, Taif 21944, Saudi Arabia; t.ismail@tu.edu.sa (T.A.I.); mmsoliman@tu.edu.sa (M.M.S.); 9 Department of Nutrition and Clinical Nutrition, Faculty of Veterinary Medicine, Zagazig University, Zagazig 44511, Egypt

**Keywords:** nano-therapy, quercetin-loaded nanoparticles, colitis, oxidative stress, inflammatory markers, immunohistochemistry

## Abstract

Oxidative stress is considered the main etiologic factor involved in inflammatory bowel disease (IBD). Integration of nanocarriers for natural therapeutic agents with antioxidant and anti-inflammatory potential is a novel promising candidate for curing IBD. Herein, the colonic antioxidant and anti-inflammatory effects of different concentrations of quercetin nanoparticles (QT-NPs) were evaluated using a dextran sulfate sodium (DSS)-induced colitis model. Following colitis induction, the efficacy and mechanistic actions of QT-NPs were evaluated by assessing lesion severity, molecular aids controlling oxidative stress and inflammatory response, and histopathological and immunohistochemistry examination of colonic tissues. Administration of QT-NPs, especially at higher concentrations, significantly reduced the disease activity index and values of fecal calprotectin marker compared to the colitic group. Colonic oxidant/antioxidant status (ROS, H_2_O_2_, MDA, SOD, CAT, GPX and TAC) was restored after treatment with higher concentrations of QT-NPs. Moreover, QT-NPs at levels of 20 mg/kg and, to a lesser extent, 15 mg/kg reduced Nrf2 and HO-1 gene expression, which was in line with decreasing the expression of iNOS and COX2 in colonic tissues. Higher concentrations of QT-NPs greatly downregulated pro-inflammatory cytokines; upregulated genes encoding occludin, MUC-2 and JAM; and restored the healthy architectures of colonic tissues. Taken together, these data suggest that QT-NPs could be a promising alternative to current IBD treatments.

## 1. Introduction

Inflammatory bowel disease (IBD), mainly including Crohn’s disease and ulcerative colitis (UC), generates a high-grade immune-associated inflammatory and oxidative process [1]. The etiology of IBD remains unclear; however, it involves a complex interaction of key factors: genetic, immunological, disturbed gut microflora and environmental factors [2]. The major clinical manifestations of IBD are gastrointestinal symptoms comprising abdominal pain, diarrhea, and bloody mucopurulent stool. Long-lasting inflammation can trigger various complications, and colorectal cancer is the most critical consequence. Consequently, IBD patients have a high risk of emerging colorectal cancer [3]. The core of the inflammatory process during IBD is formation of reactive oxygen species (ROS) and reactive nitrogen species (RNS), thought to provoke the overproduction of pro-inflammatory cytokines that results in excessive production of ROS/RNS and oxidative/nitrosative stress intensification [4]. IBD is characterized by macrophage and neutrophil influx, which in turn promotes cytokines, free radicals and proteolytic enzymes production, eliciting inflammation and ulceration [5]. Nuclear factor erythroid 2-related factor 2 (Nrf2), a significant factor in cells’ oxidative stress response, regulates the expression of antioxidant proteins and phase II detoxification enzymes [6]. Colitis is associated with the Nrf2 signaling pathways influencing its target genes and their expression (Lu et al., 2016). The Nrf2/HO-1 signaling pathway mainly involved in chemically induced colitis and its activation is considered a promising new therapeutic approach [7,8,9]. Until now, the disease has been considered incurable, and therapies are primarily pursuing the inflammation symptoms [10]. Moreover, the current pharmaceutical treatments are dangerous or ineffective for long-term use and are associated with an unfavorable side consequence; hence, promising new strategies considering substances with antioxidant curative properties are continually sought. These substances must have a high safety profile as well as control the progression of disease positively. In this context, antioxidant therapy can be considered a crucial regulatory factor of colitis. Furthermore, both patients and healthcare specialists are increasingly interested in investigating the impact of nutritional therapy on sustaining the remission of IBD [11]. Diet may positively influence the development of colitis through a variety of biological mechanisms, such as gut microbiome modifications, antigen presentation, and functions of the epithelial barrier and mucosal immune system [12]. Among these important dietary natural agents is quercetin, which is the main polyphenolic flavonoid in a variety of vegetables and fruits [13] and acts as a strong antioxidant with powerful scavenging capacity in biological systems for protecting the tissues from free radicals [14,15]. Additionally, quercetin is able to boost the formation of cellular antioxidants [16]. It is also used for its antimicrobial, anticancer, antidiabetic and antiatherosclerosis activities and for curing metabolic, inflammatory and cardiovascular diseases [17]. Many mechanisms are involved in scavenging ROS by quercetin via eliminating O_2_^−^ [18], NO^−^ [19] and ONOO^−^ [20]. When considering the impact of quercetin on intestinal inflammation, many studies have revealed that quercetin has intestinal anti-inflammatory and antioxidant effects [21,22]. Meanwhile, the fact that quercetin can be absorbed in the stomach and small intestine and rapidly metabolize or suffer strong degradation may hinder its access to the colon at desired concentrations to display local antioxidant and anti-inflammatory effects, thus restricting its potential use for IBD management [23]. Hence, the engineering of innovative dietary formulations targeting nanoparticles (NPs) offers functional food ingredients with various novel features that enhance their bioavailability, quality and safety together with increasing their stability and protecting them from intestinal degradation [24,25,26,27]. The fate of nanoparticle-based functional ingredients in the gastrointestinal tract greatly varies from that of ingredients that have larger particles owing to their higher surface area with greater motion proficiency, enabling them to easily penetrate the biological barriers, including intestinal epithelial cells [28,29]. For instance, quercetin-loaded microcapsules have been found to reduce experimental colitis and show higher efficiency than free quercetin [30], owing to their effective bioavailability and solubility [31]. Since quercetin nano formulation can display novel promising properties that accelerate its bioavailability, it is hypothesized that the application of a nanotechnology-based quercetin delivery system may augment its functional properties [32]. Thus, in this context, we proposed that the combination of quercetin with specific nanoparticles (NPs) will boost their ongoing function in IBD therapy. The overall aims of this study were to synthesize new quercetin-based NPs that will result in the better efficacy of quercetin therapy against IBD and to investigate their antioxidant and anti-inflammatory effects. Therefore, a DSS-induced colitis model was employed in our experiments to prove the possibility of creating “natural” nano vectors from different quercetin concentrations that proficiently mediate in vivo therapy for human colitis.

## 2. Materials and Methods

### 2.1. Synthesis and Characterization of Quercetin Nanoparticles

**Figure 1:**
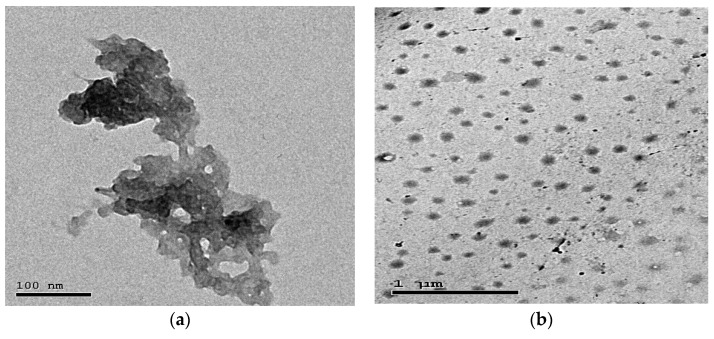
Transmission electron microscopy (**a**,**b**) of quercetin-loaded nanoparticles.

Chitosan nanoparticles were prepared by electrostatic gelation of chitosan (9012-76-4, Sigma Aldrich, St. Louis, MO, USA) as performed according to [24]. Briefly, 2 mg of chitosan was dissolved in 1% glacial acetic solution, stirred overnight at 500 rpm and centrifuged at 9000 rpm for 20 min. Then, 4 mL of sodium tripolyphosphate (TPP) solution (7758-29-4, Sigma Aldrich, St. Louis, MO, USA) was poured into 10 mL of chitosan solution. Lastly, 3 mg of quercetin (849061-97-8, Sigma Aldrich, St. Louis, MO, USA) was loaded into the prepared chitosan nanoparticles solution, and then, TPP solution was added under magnetic stirring (2000 rpm), and the prepared mixture was then centrifuged and lyophilized. The physicochemical properties of QT-NPs were confirmed by transmission electron microscopy (Figure 1a,b).

### 2.2. Animals, DSS-Induced Colitis Model and Treatment Protocol

Male Wistar rats aged 6–7 weeks with an average body weight of 206.92 ± 7 g were kept in an experimental animal house and maintained under controlled environmental conditions at relative humidity of 56 ± 4% and temperature of 23 ± 1 °C. Rats were acclimated for 2 weeks prior to the experimental period and given free access to water and food. Colitis was induced by administration of DSS (3%) (MW 36,000–50,000, MP Biomedicals, Southern, CA, USA) [33] in the fresh drinking water for 7 successive days. After induction of colitis, all rats received quercetin-loaded nanoparticles (QTNPs) for 2 weeks. Experimental rats were distributed randomly into 5 groups in separate cages based on the body weight, as follows. Control group rats were fed standard diets. The Colitis model included four groups: the DSS group; the DSS + QTNPsI group: rats received 10 mg/kg body weight of QTNPs after induction of colitis; the DSS + QTNPsII group: rats received 15 mg/kg body weight of QTNPs after induction of colitis; and the DSS + QTNPsIII group: rats received 20 mg/kg body weight of QTNPs after induction of colitis. All experimental conditions of this study were in agreement with the recommendations and regulations endorsed by the Institutional Animal Care and Use Committee (ZU-IACUC/2021), Faculty of Veterinary Medicine, Zagazig University, Zagazig, Egypt.

### 2.3. Assessment of Colitis Clinical Signs and Lesion Severity

Daily observations were performed during the experimental period by monitoring body mass loss and physical activity. The length of colon segment, spleen weight, severity of diarrhea, and rectal bleeding were evaluated and scored as described by standard protocols [34]. The scores of disease activity index (DAI) varied from 0 (for healthy rats) to 12 (for rats exhibiting severe colitis) using the scoring system in Table 1 according to [35]. The total disease severity was estimated with a clinical scoring system as the % weight loss, rectal bleeding and stool consistency scored from 0 to 4. The estimated values were expressed for each animal, and the sum of the 3 values corresponded to the DAI. Fecal matter was employed for rectal bleeding evaluation (from occult blood to gross bleeding). Meanwhile, diarrhea was confirmed by presence of mucus on fecal matter.

### 2.4. Sampling Procedures

All rats under experimental conditions were anesthetized via intravenous ketamine hydrochloride (30 mg/kg BW) and then euthanized by cervical dislocation. Blood was collected in heparinized tubes for hematological indices and for serum separation (centrifuged at 4 °C and 4000 rpm for 10 min). Fecal samples were used for estimating fecal calprotectin marker. The colon specimens (the distal 10 cm portion of the colon) were removed and washed with PBS to remove any fecal deposits. The collected colon specimens were transmitted directly to be stored at −80 °C for gene expression (RT-qPCR). The other colon specimens were homogenized, and then the homogenates were used for antioxidants and oxidative stress biomarkers assessment. For histopathological and immunohistochemical examination, the colon tissues were fixed in 10% formalin buffer.

### 2.5. Hematological and Serum Assessment

Hematological indices (red blood cell counts (RBC) and hemoglobin (Hb) concentration) were determined as defined by [36]. The serum concentrations of ALT, AST, urea and creatinine were assessed via commercial kits (Sigma-Aldrich, MAK080, MAK006, MAK052, MAK055, respectively). C-reactive protein (CRP) concentrations were evaluated by standard kit (AG723-M, Sigma-Aldrich).

### 2.6. Profiling of Fecal Calprotectin

Fecal samples were collected 2, 4, 6, 8, 10, 12, and 14 days after colitis induction, suspended in extraction buffer, and homogenized for 25 min. One milliliter of the homogenate was centrifuged for 15 min. The resulting supernatant was used to determine the level of fecal calprotectin by ELISA assay [37].

### 2.7. Enzyme Linked Immunosorbent Assay (ELISA) for Cytokines

Colon IL-6, IL-10 TNF-α, and *IFN-γ* levels were assessed following the manufacturer’s instructions via Thermo Fisher cytokine commercial ELISA kits (BMS625, BMS629, BMS622 and BMS621, respectively).

### 2.8. Profiling of Colonic Myeloperoxidase and Nitric Oxide

The activity of myeloperoxidase (MPO) in the colonic samples was evaluated according to [38]. Briefly, colonic samples were weighed, then homogenized in 10 mL potassium phosphate buffer, pH 6.0, hexadecyl trimethyl ammonium bromide, and ethylene acetic acid. In the later step, the prepared homogenates were centrifuged for 20 min at 2000 rpm, and the supernatant was utilized for MPO assay by BioVision kits (E4581-100). MPO values were expressed as units/g (U/g) of tissue wet weight. Nitric oxide (NO) in supernatant of colon homogenates was determined corresponding to [39] with minor modification, exchanging zinc sulfate as a substitute for ethanol for the proteins precipitation. Absorbance was evaluated at 540 nm, and the statistics were quantified as nmol/g colonic tissue.

### 2.9. Assessment of Colonic Oxidants/Antioxidants Status of Colonic Tissues

The colon tissue homogenates were used for the malondialdehyde (MDA) and total antioxidant capacity (TAC) estimation (CAT. NO. MBS508035 and CAT. NO. MBS169313), respectively, following manufacturer’s instructions. ELISA kits (MyBioSource; San Diego, CA, USA) were used for estimation of superoxide dismutase (SOD, CAT. NO. MBS036924), catalase (CAT. NO. MBS006963), and glutathione peroxidase (GPX, CAT. NO. MBS028183). The colon ROS content was determined via a specific enzyme-linked immunosorbent assay (ELISA) kit (MBS039665, MyBioSource; San Diego, CA, USA) following the manufacturer’s instructions. Colon hydrogen peroxide (H_2_O_2_) levels were estimated according to the procedures previously detailed by [40], and their values were expressed as μmol/g of tissue.

### 2.10. RNA Extraction and Quantitative Real-Time PCR

Colonic JAM, mucin, occludin, nrf-2, HO-1, TLR-4, CD-4 and CD-8 expression levels were evaluated. Initially, total RNA was extracted from the colon tissue using a Trizol Reagent (Thermo Fisher Scientific; Waltham, MA, USA) in line with the manufacturer’s instructions. Then, two-step real-time PCR was adopted for gene expression assessment. Briefly, cDNA synthesis was performed using a Maxime RT PreMix (Oligo (dT)15 Primer) cDNA Synthesis Kit (Cat. No. 25081, iNtRON Biotechnology Co., Seongnam, Korea), followed by real-time PCR. The forward and reverse primer sequences used in the RT-PCR analysis are described in Table 2. The RT-qPCR reaction was analyzed by a Rotor-Gene Q2 plex (Qiagen Inc., Valencia, CA, USA) using 2 × SYBR green PCR master mix (12.5 μL) (QuantiTect) and 1 μL of each primer (10 pmol/mL) for each gene, cDNA (2 μL), and RNase free water (8.5 μL) in a total volume of 25 μL. Relative fold changes in the expression of target genes were calculated using the comparative 2^−ΔΔCt^ (where Ct is the cycle threshold) method described by Livak and Schmittgen [41]. The β-actin gene was used as an internal control to normalize the expression levels of the target genes.

### 2.11. Histopathological Analysis

After decapitation, tissue samples from the distal colon were collected, immediately fixed in 10% neutral buffered formalin for 24 h, trimmed into 3.0 mm thick tissue sections, washed in distilled water, dehydrated in graded ethyl alcohol (70–100%), cleared in two changes of xylene (1 h each), impregnated (12 h) and embedded in paraffin-beeswax mixture (90% paraffin and 10% beeswax), and cut using microtome (RM2125 RTS, Leica Deer Park, IL 60010 United States). Then, obtained tissue sections (5 μm) were processed for staining with hematoxylin and eosin stains, and mounted in dibutylphthalate polystyrene xylene (DPX) [42,43,44]. The slides were examined microscopically, and the histopathological lesions were recorded.

### 2.12. Immunohistochemical Detection of iNOS and COX-2

Successive tissue sections of 5.0 μm thickness were obtained from the formalin-fixed paraffin-beeswax-embedded tissue blocks for iNOS (anti-iNOS antibody (ab3523), 1:20 dilution) and COX-2 (1:1000 dilution; anti-COX2/cyclooxygenase 2 antibody (ab15191), Cambridge, UK, abcam, goat anti-rabbit IgG H&L (HRP) secondary antibody (ab205718) and 3,30-Diaminobenzidine chromogen (DBA) (Abcam, Cambridge, UK)), following the Avidin-Biotin-Peroxidase Complex (ABC) protocol developed by Hsu et al. [45]. Negative control sections were prepared by using phosphate buffer saline as a substitute for the iNOS and COX-2 primary antibodies. The cells’ nuclei were counterstained with Mayer’s hematoxylin [46]. The immunostained sections were analyzed with light microscopy, and the staining intensity was assessed by ImageJ software (v 1.53, National Institutes of Health, Bethesda, MD, USA) as previously described by Jensen [47].

### 2.13. Statistical Analysis

Statistical assessments of the experimental data were accomplished by SPSS^®^ Statistics program version 22 (SPSS Inc., Chicago, IL, USA). The validation of statistical tests was assessed by normality of variance and homogeneity using Shapiro–Wilk and Levene tests, respectively. The data were examined by one-way analysis of variance (ANOVA), then variations among groups were evaluated by Tukey post hoc test. All experimental variables were expressed as means ± standard errors (SE). Statistically significant levels were anticipated at *p*-values 0 < 0.05. Differences were considered significant when *p* < 0.05. All graphs were designed via the GraphPad Prism software (Version 8, GraphPad Software Inc., San Diego, CA, USA).

## 3. Results

### 3.1. Liver and Kidney Functions and Hematological Indices

As shown in Table 3, hematological indices, including RBC counts and Hb concentrations, were affected in colitic groups, as we noted that there was a significant decrease (*p* < 0.05) in RBC counts and Hb concentration in the colitic non-treated group when compared with the non-colitic control group. However, the treatment with QT-NPs, especially at higher concentrations, could efficiently reverse the DSS-induced anemic index in a dosage-related manner. The activities of hepatic enzymes ALT and AST were significantly elevated in the serum of colitic non-treated rats compared with those assessed in the non-colitic group. In contrast, QT-NP administration significantly reversed these elevations. Additionally, serum levels of urea and creatinine in kidney function tests were significantly increased (*p* < 0.05) in the colitic rat model. Remarkably, oral administration of QT-NPs after induction of colitis recovered kidney function to levels approximately similar to those of the control non-colitic group.

### 3.2. Impacts of QT-NPs Therapy on Elevation of Colitis Symptoms

**Figure 2:**
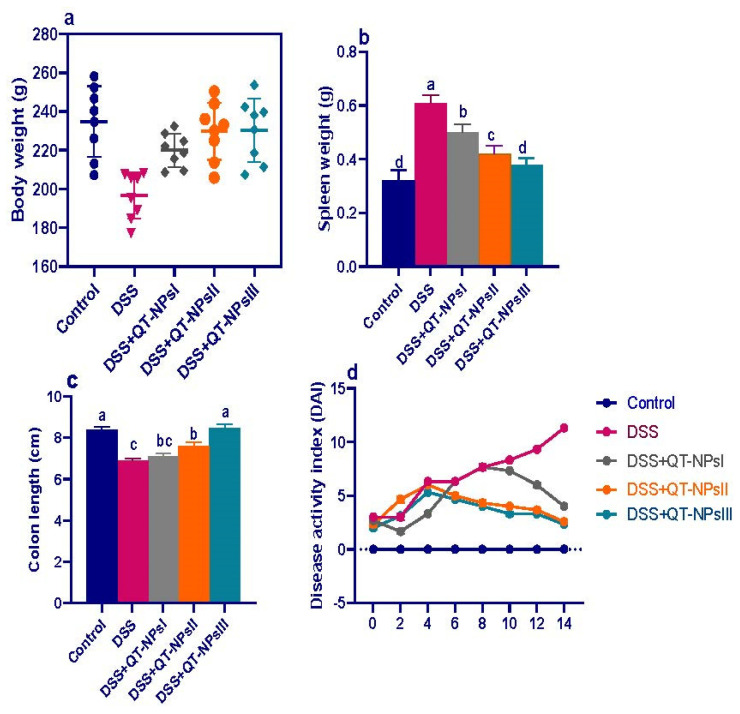
Impacts of quercetin-loaded nanoparticles (QT-NPs) therapy on colitic signs. (**a**) Body weight gain. (**b**) Spleen weight, (**c**) colon length, (**d**) disease activity index score. Control: healthy non-colitic group (rats orally gavaged with PBS); colitic groups: DSS (rats orally gavaged with dextran sodium sulphate), DSS + QT-NPsI (rats orally gavaged with DSS and quercetin-loaded nanoparticles (QT-NPs at the level 10 mg/kg body weight for 14 days)), DSS + QT-NPsII (rats orally gavaged with DSS and quercetin-loaded nanoparticles (QT-NPs at the level 15 mg/kg body weight for 14 days)), DSS + QT-NPsIII (rats orally gavaged with DSS and quercetin-loaded nanoparticles (QT-NPs at the level 20 mg/kg body weight for 14 days)). All groups were orally gavaged by 3% DSS. ^a–d^ Means of the rows with different letters were significantly different among groups (*p* < 0.05). Control: healthy non-colitic group (rats orally gavaged with PBS); colitic groups: DSS (rats orally gavaged with dextran sodium sulphate), DSS + QT-NPsI (rats orally gavaged with DSS and quercetin-loaded nanoparticles (QT-NPs at the level 10 mg/kg body weight for 14 days)), DSS + QT-NPsII (rats orally gavaged with DSS and quercetin-loaded nanoparticles (QT-NPs at the level 15 mg/kg body weight for 14 days)), and DSS + QT-NPsIII (rats orally gavaged with DSS and quercetin-loaded nanoparticles (QT-NPs at the level 20 mg/kg body weight for 14 days)). All groups orally gavaged by 3% DSS.

The attenuating effects of different concentrations of QT-NPs on the severity of clinical signs after induction of colitis in rats are illustrated in Figure 2. Weight loss was more remarkable in rats suffering from colitis (up to 14%), while colitic rats receiving QT-NPsIII regained their body weight (Figure 2a). An increased spleen weight was noted in DSS colitic rats, while a non-significant change in spleen weight was found in groups receiving QT-NPsIII (Figure 2b). Notably, rats treated with QT-NPsIII exhibited the highest (*p* < 0.05) significant colon length (8.47 ± 0.19 cm vs. 6.89 ± 0.12 cm in the DSS group) (Figure 2c). Rats from the DSS-induced colitis model displayed elevated DAI scores as evaluated by measuring weight loss and the presence of rectal bleeding and diarrhea. During the progression of colitis, colitic rats receiving higher concentrations of QT-NPs showed reduced DAI scores (Figure 2d). Remarkably, rats who were in the DSS-induced colitis model and treated with QT-NPsIII were kept alive during the experimental period.

### 3.3. Evaluation of Histopathological Damage after Induction of Colitis in Response to QT-NPs Therapy

**Figure 3:**
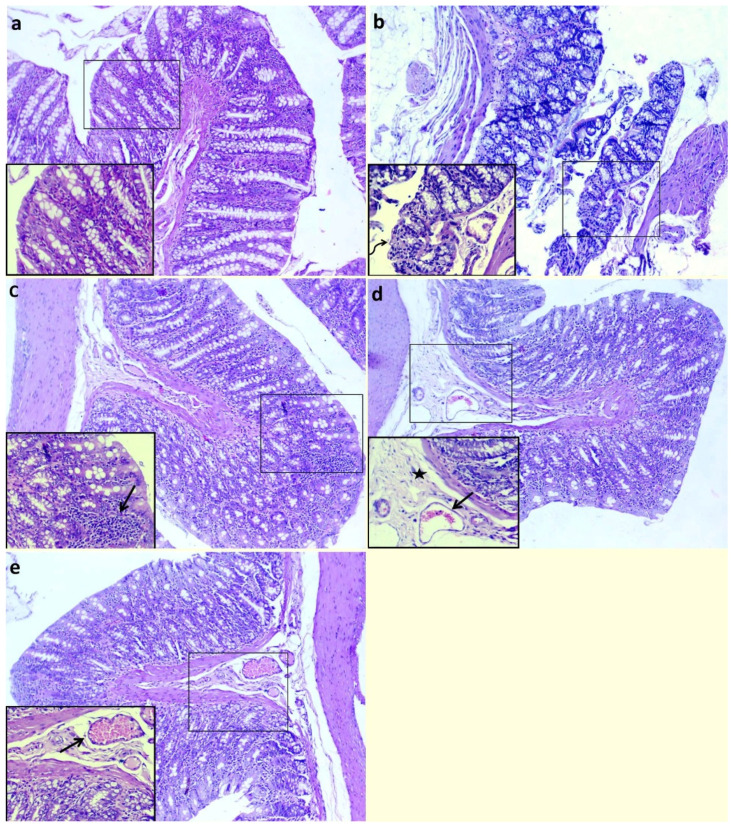
Impacts of quercetin-loaded nanoparticles (QT-NPs) therapy on histopathological lesion. Control: healthy non-colitic group (rats orally gavaged with PBS, (**a**)) showing normal histological structures of columnar epithelial lining mucosa with goblet cells, submucosa, muscular layer and serosal layer. Colitic groups: DSS (rats orally gavaged with dextran sodium sulphate, (**b**)) showing necrotic lamina epithelialis (arrow) represented by coagulative necrosis of epithelium admixed with leukocytic infiltrates (curved arrow); DSS + QT-NPsI (rats orally gavaged with DSS and quercetin-loaded nanoparticles (QT-NPs at the level 10 mg/kg body weight for 14 days), (**c**)) showing preserved structures of colon layers with presence of inflammatory cells infiltrates within lamina propria; DSS + QT-NPsII (rats orally gavaged with DSS and quercetin-loaded nanoparticles (QT-NPs at the level 15 mg/kg body weight for 14 days), (**d**)) showing edema (star) with mildly dilated blood vessels (arrow) within submucosal layer; and DSS + QT-NPsIII (rats orally gavaged with DSS and quercetin-loaded nanoparticles (QT-NPs at the level 20 mg/kg body weight for 14 days), (**e**)) showing preserved structures of mucosa, submucosa, musculosa and serosa in addition to dilated submucosal blood vessels (arrow). All groups orally gavaged by 3% DSS. Magnification: large micrographs are 100× and smaller ones in the lower left corner are 400×.

Examination of histopathological pictures is shown in Figure 3. The non-colitic control group showed normal histological structures of columnar epithelial lining mucosa with goblet cells, submucosa and muscular and serosal layers (Figure 3a). The colitic non-treated group showed necrotic lamina epithelialis in some examined sections, which was represented by coagulative necrosis of the epithelium admixed with leukocytic infiltrates in addition to dilated submucosal blood vessels (Figure 3b). Moreover, the group treated with QT-NPsI revealed preserved structures of colon layers with the presence of inflammatory cell infiltrates within the lamina propria (Figure 3c). The group treated with QT-NPsII revealed edema with mildly dilated blood vessels within the submucosal layer (Figure 3d). The group treated with QT-NPsIII displayed apparent normal mucosa, submucosa, musculosa and serosa in addition to dilated submucosal blood vessels (Figure 3e).

### 3.4. Assessment of Fecal Calprotectin Levels in Response to QT-NPs Therapy

**Figure 4:**
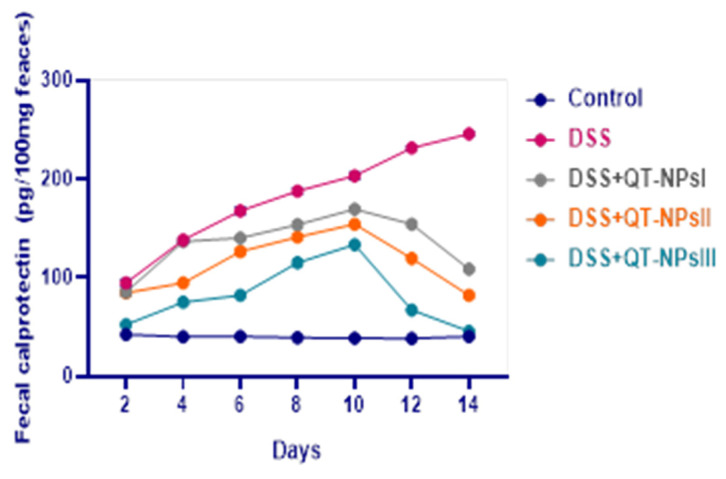
Impacts of quercetin-loaded nanoparticles (QT-NPs) therapy on fecal calprotectin levels post DSS induction. Control: healthy non-colitic group: (rats orally gavaged with PBS); Colitic groups: DSS (rats orally gavaged with dextran sodium sulphate), DSS + QT-NPsI (rats orally gavaged with DSS and quercetin-loaded nanoparticles (QT-NPs at the level 10 mg/kg body weight for 14 days)), DSS + QT-NPsII (rats orally gavaged with DSS and quercetin-loaded nanoparticles (QT-NPs at the level 15 mg/kg body weight for 14 days)), and DSS + QT-NPsIII (rats orally gavaged with DSS and quercetin-loaded nanoparticles (QT-NPs at the level 20 mg/kg body weight for 14 days)). All groups orally gavaged by 3% DSS.

Levels of fecal calprotectin in fecal samples following induction of colitis in different groups are represented in Figure 4. In the colitic non-treated group, fecal calprotectin levels were raised from d 2 to d 14, whereas all groups receiving QT-NPs saw their levels reduced from d 10 in a dose-dependent manner. Interestingly, at d 14, the levels of fecal calprotectin in QT-NPsIII were restored and reached the fecal calprotectin level of the control non-colitic group.

### 3.5. Assessment of Oxidative Stress Biomarkers and Antioxidant Defense in Colon

**Figure 5:**
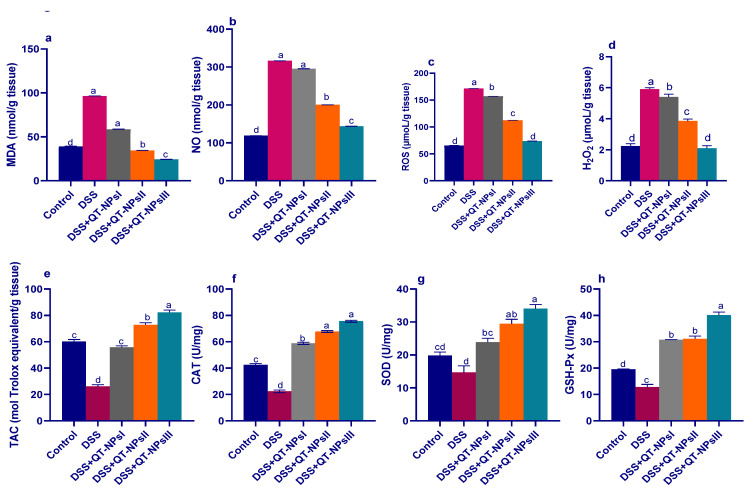
Impacts of quercetin-loaded nanoparticles (QT-NPs) therapy on MDA (**a**), nitric oxide (NO) (**b**), ROS (**c**), H_2_O_2_ (**d**), total antioxidant capacity (TAC) (**e**), CAT (**f**), SOD (**g**), and GSH-Px (**h**). Fourteen days post DSS induction. Control: healthy non-colitic group (rats orally gavaged with PBS); colitic groups: DSS (rats orally gavaged with dextran sodium sulphate), DSS + QT-NPsI (rats orally gavaged with DSS and quercetin-loaded nanoparticles (QT-NPs at the level 10 mg/kg body weight for 14 days)), DSS + QT-NPsII (rats orally gavaged with DSS and quercetin-loaded nanoparticles (QT-NPs at the level 15 mg/kg body weight for 14 days)), and DSS + QT-NPsIII (rats orally gavaged with DSS and quercetin-loaded nanoparticles (QT-NPs at the level 20 mg/kg body weight for 14 days)). All groups orally gavaged by 3% DSS. ^a–d^ Means of the rows with different letters were significantly different among groups (*p* < 0.05). Control: healthy non-colitic group (rats orally gavaged with PBS); colitic groups: DSS (rats orally gavaged with dextran sodium sulphate), DSS + QT-NPsI (rats orally gavaged with DSS and quercetin-loaded nanoparticles (QT-NPs at the level 10 mg/kg body weight for 14 days)), DSS + QT-NPsII (rats orally gavaged with DSS and quercetin-loaded nanoparticles (QT-NPs at the level 15 mg/kg body weight for 14 days)), and DSS + QT-NPsIII (rats orally gavaged with DSS and quercetin-loaded nanoparticles (QT-NPs at the level 20 mg/kg body weight for 14 days)). All groups orally gavaged by 3% DSS.

As described in Figure 5, levels of ROS, NO, MDA and H_2_O_2_ were significantly reduced (*p* < 0.05) with increasing levels of QT-NPs. Conversely, the reduced TAC capacities in the colitic non-treated group were significantly elevated in the colitic groups receiving QT-NPs in a dose-dependent manner. Prominently, administration of different levels of QT-NPs after colitis induction greatly increased (*p* < 0.05) the activities of GSH-Px, SOD and CAT enzymes in colonic tissues.

### 3.6. Quantification of Inflammatory Biomarkers in Colon by ELISA

**Figure 6:**
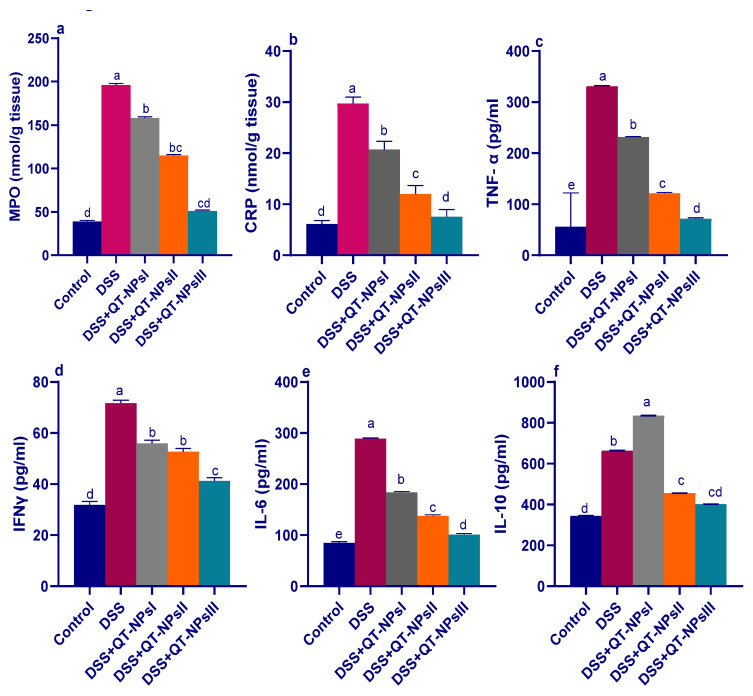
Impacts of quercetin-loaded nanoparticles (QT-NPs) therapy on MPO: myeloperoxidase (**a**), CRP: C-reactive protein (**b**), TNF-α: tumor necrosis factor alpha (**c**), IFNγ: Interferon gamma (**d**), IL-6 and IL-10: interleukin (**e**,**f**) 14 days post DSS induction. Mean values with different letters in the same row differ significantly at *p* < 0.05, SE: standard error. ^a–e^ Means of the rows with different letters were significantly different among groups (*p* < 0.05). Control: healthy non-colitic group (rats orally gavaged with PBS). Colitic groups: DSS (rats orally gavaged with dextran sodium sulphate), DSS + QT-NPsI (rats orally gavaged with DSS and quercetin-loaded nanoparticles (QT-NPs at the level 10 mg/kg body weight for 14 days)), DSS + QT-NPsII (rats orally gavaged with DSS and quercetin-loaded nanoparticles (QT-NPs at the level 15 mg/kg body weight for 14 days)), and DSS + QT-NPsIII (rats orally gavaged with DSS and quercetin-loaded nanoparticles (QT-NPs at the level 20 mg/kg body weight for 14 days)). All groups orally gavaged by 3% DSS.

As shown in Figure 6, after DSS exposure, the highest MPO activities (*p* < 0.05) in colon tissues were noted in the colitic groups, while this raised level was significantly lowered in the QT-NPsIII group (Figure 6a). Of note, the levels of CRP were increased and reached their peak in the colitic non-treated group, while the QT-NPsIII group displayed nearly no significant change in CRP levels when compared to the non-colitic group. Efficacy of administration of QT-NPs toward inflammatory response in the colon was confirmed by cytokine quantification using ELISA kits, as displayed in Figure 6c–f. The maximum inflammatory response was found in the DSS-induced colitis model, as shown by higher detected levels (*p* < 0.05) of IL-6, TNF-α, IFN-γ, and IL-10 cytokines. Their levels were significantly decreased after increasing the levels of received QT-NPs.

### 3.7. Efficacy of QT-NPs Therapy on the Expression of Tight Junction-Related Genes in Colitic Rats

**Figure 7:**
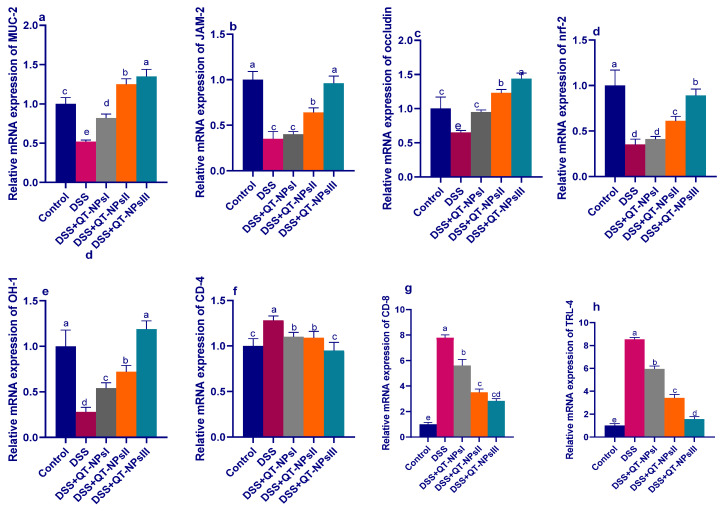
Impacts of quercetin-loaded nanoparticles (QT-NPs) therapy on mRNA expression of tight junction-related genes mucin-2 (MUC-2) (**a**), junction adhesion molecule (JAM) (**b**), occludin (**c**), nuclear factor erythroid 2-related factor 2 (NRF2)-Kelch-like erythroid cell-derived protein (**d**), heme oxygenase-1 (Hmox1, HO-1) (**e**), gene encoding the CD4 membrane glycoprotein of T lymphocytes (**f**), gene encoding the CD8 membrane glycoprotein of T lymphocytes (**g**), toll-like receptor-4 (TLR-4) (**h**) 14 days post DSS induction. Mean values with different letters in the same row differ significantly at *p* < 0.05, SE: standard error. ^a–e^ Means of the rows with different letters were significantly different among groups (*p* < 0.05). Control: healthy non-colitic group (rats orally gavaged with PBS); colitic groups: DSS (rats orally gavaged with dextran sodium sulphate), DSS + QT-NPsI (rats orally gavaged with DSS and quercetin-loaded nanoparticles (QT-NPs at the level 10 mg/kg body weight for 14 days)), DSS + QT-NPsII (rats orally gavaged with DSS and quercetin-loaded nanoparticles (QT-NPs at the level 15 mg/kg body weight for 14 days)), and DSS + QT-NPsIII (rats orally gavaged with DSS and quercetin-loaded nanoparticles (QT-NPs at the level 20 mg/kg body weight for 14 days)). All groups orally gavaged by 3% DSS.

As illustrated in Figure 7, among all DSS-induced groups, the expression levels of the tight junction-encoding genes were markedly decreased in the DSS-induced and non-treated group, unlike the control non-colitic group. In contrast, expressions of MUC-2 and occludin achieved their peaks in the QT-NPsIII colitic group (increased by 1.25- and 1.44-fold, respectively, vs. the non-colitic group). Among all colitic groups, the higher expression of JAM was more prominent in the QT-NPsIII and QT-NPsII groups.

### 3.8. Efficacy of QT-NPs Therapy on the Expression of Tight Junction-Related Genes in Colitic Rats

As illustrated in Figure 7, among all DSS-induced groups, the expression levels of the tight junction-encoding genes were markedly decreased in the DSS-induced and non-treated group, unlike the control non-colitic group. In contrast, expression of MUC-2 and occludin achieved their peaks in the QT-NPsIII colitic group (increased by 1.25 and 1.44-fold, respectively, vs. levels in the non-colitic group). Among all colitic groups, the higher expression of JAM was more prominent in the QT-NPsIII and QT-NPsII groups.

### 3.9. Expression Dynamics of Inflammatory Mediators and Nrf2 and HO-1

The mRNA expression levels of the *TLR-4*, *CD-4*, *CD-8*, *Nrf2* and *HO-1* genes are shown in Figure 7. After induction of experimental colitis, the relative expression levels of *CD-4*, *CD-8* and *TLR-4* (Figure 7f–h) were significantly downregulated with increasing concentrations of QT-NPs, unlike the colitic non-treated group, which exhibited higher expression levels of these genes. Another remarkable observation to emerge from the results of the analyses was the upregulation in expression of the Nrf2 and HO-1 genes, particularly with higher concentrations of QT-NPs relative to the control colitic group (Figure 7d,e).

### 3.10. Immunohistochemical Detection of iNOS and COX2 in the Colon

**Figure 8:**
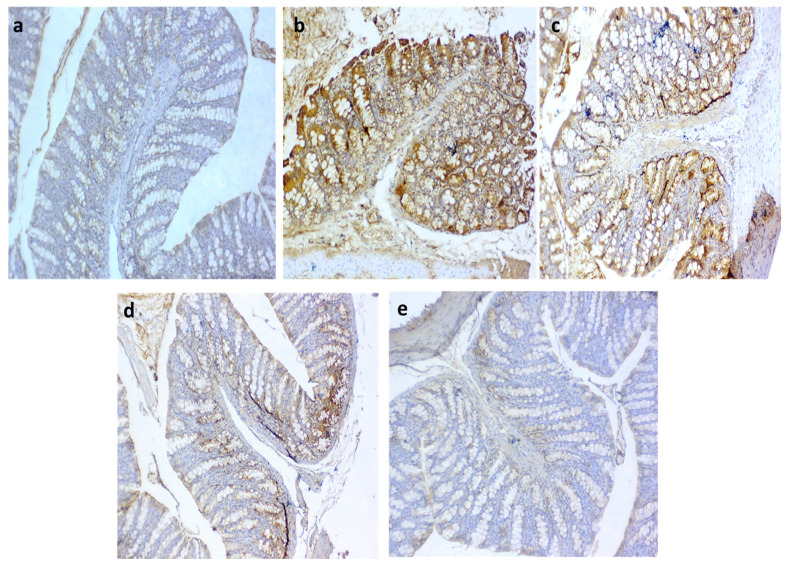
Photomicrographs of immunoexpression for iNOS in colon sections showing (**a**) negative control with no positive reaction; (**b**) positive control group with intense immunopositive reactions of anti iNOS in DSS group; (**c**) moderate number of immunopositive cells in QT-NPsI group; (**d**) weak stained cells in few numbers in colon epithelium in QT-NPsII group; (**e**) no immunoreactive cells in QT-NPsIII group. IHC counterstaining with Mayer’s hematoxylin. Scale bar 100 µm.

**Figure 9:**
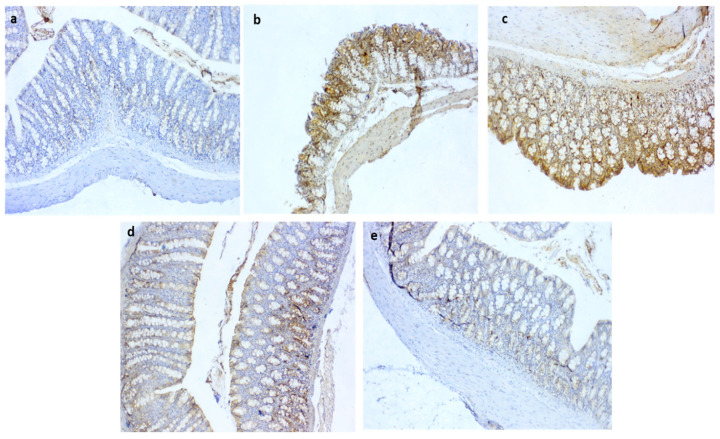
Photomicrographs of immunoreactivity for COX2 in colon sections showing (**a**) negative control with negative stained cells; (**b**) +ve group with markedly increased immunostaining of cells; (**c**) moderate COX2 expression in QT-NPsI group; (**d**) few numbers of immunolabelled cells for COX2 in QT-NPsII group; (**e**) few immunostaining of cells in QT-NPsIII group. IHC counterstaining with Mayer’s hematoxylin. Scale bar 100 µm.

Regarding iNOS expression, it was noted that the colitic non-treated group showed intense immunopositive reactions of anti-iNOS (Figure 8b) in the colonic sections. In contrast, moderate to fewer numbers of immunopositive cells were seen in the QT-NPsI and QT-NPsII groups (Figure 8c,d, respectively). Remarkably, the non-colitic and QT-NPsIII groups showed no staining reactions within the epithelial lining of colon mucosa against the iNOS marker (Figure 8a,e, respectively). The expression of COX2 after colitic induction is presented in Figure 9, and unlike the non-colitic group, it showed negative staining for COX2 expression (Figure 9a). Meanwhile, the immunolabelled cells of the colon decreased with increasing concentrations of QT-NPs (Figure 9c–e). Conversely, the colitic non-treated group displayed overexpression of COX2 (Figure 9b) in the colonic sections.

## 4. Discussion

Excessive production of the superoxide radical anion (O_2_^−•^) and H_2_O_2_ together with reduced levels of antioxidants in the inflamed colon are correlated with the pathogenesis of IBD [48]. The disease conditions give rise to physiological alterations associated with oxidative stress caused by an imbalance between immune responses and the production of nitrogen species/reactive oxygen species and antioxidant defenses [49]. Recently, the use of natural compounds with antioxidant capacity was found to counteract cell damage by scavenging these harmful free radicals and supporting the prevention of the disease [50,51]. Quercetin is the main polyphenolic flavonoid showing excellent antioxidant and scavenging capacity in addition to immunostimulant properties [15,52]. However, certain limitations that restrict its application, such as poor bioavailability, instability, aqueous solubility and permeability, still exist. The emergence of nanotechnology has provided the opportunity to incorporate quercetin into nano-delivery systems that accelerate its targeted delivery to the inflamed sites and control its release in the GIT [53,54,55]. In reviewing the earlier literature, no data were targeted on the prospective impacts of QT-NPs on treating IBD.

In the current study, DSS-induced rats showed a prominent weight loss (14% loss of body weight) and altered stool consistency with the consequence of bloody diarrhea that agreed with [56]. There is a correlation between colitis and enlargement of the spleen in animals as it is considered an organ index of inflammation [57]. Colon length is inversely correlated with the severity of DSS-induced colitis [58]. Interestingly, our study demonstrated that oral administration of QT-NPs resulted in restoration of lost body weight and reduced spleen weight in the DSS-induced rats. Notably, the colon length of the QT-NP-treated group did not differ greatly from the control non-DSS-induced rats. In accordance, the mechanisms and therapeutic effects of orally administered quercetin on treating induced colitis were stated [30]. Additionally, the body weight loss in colitic-induced rats was restored after dietary supplementation with 30 mg/kg quercetin [59]. The DAI score, a crucial clinical manifestation of IBD, is a vital indicator used to assess colonic damage [60]. Herein, the colitic-induced group showed diarrhea, bloody stool and weight loss that contributed to a higher DAI. In contrast, these clinical symptoms were greatly diminished by increasing the level of QT-NPs, indicating that experimentally induced colitis was efficiently reduced by treatment with QT-NPs. Similarly, the use of natural antioxidants such as Garcinia Kola nuts was able to decrease the symptom severity of colitis in mice [61]. In addition, DSS-induced weight loss and histopathological alterations were significantly restored by flavonoid treatment [62,63,64]. FC is a pro-inflammatory protein, and it plays a significant role in sites of inflammation by triggering the toll-like receptors [65].

Levels of fecal calprotectin (FC) are considerably increased during active periods of inflammation, and it is an important indicative biomarker of disease severity, specifically in IBD [66]. This elevation in fecal calprotectin signifies the migration of neutrophils into the intestinal mucosa as a consequence of intestinal injury [67]. An elevation in FC was detected in colitic non-treated rats, unlike the group treated with multi-strain-loaded nanoparticles [25]. Remarkably, the current study showed that the positive impacts of QT-NPs on FC reduction were more prominent when their levels were increased after the induction of colitis. The actual mechanism underlying these boosted roles of QT-NPs can be attributed to several benefits associated with the reduction in size of the particles to the nanometric scale, including active ingredient release and enhanced bioavailability and dispersion ability, and consequently exerting their beneficial role in attenuating the inflammation [68]. Notably, rats suffering from colitis also exhibited anemia as evidenced by reduced RBC counts and Hb levels and disturbances of hepatic and kidney functions that agreed with [69,70]. Conversely, administration of QT-NPs prominently restored the RBCs and Hb levels after induction of colitis. Moreover, QT-NPs protected liver and kidney tissues from damage caused by oxidative stress, thus preventing the leakage of hepatic enzymes and kidney waste products into circulation.

The gastrointestinal tract is a key source of ROS [71]. Excessive ROS production can activate lipid peroxidation of cell membranes and cause subsequent tissue damage. In particular, the colon produces more endogenous ROS than the small intestine, and this overproduction of ROS exceeds the colonic antioxidant enzyme capacity to mitigate oxidative DNA damage [72]. Inflammation is linked to the recruitment and stimulation of mucosal phagocytes, which release higher levels of ROS, and such uncontrolled ROS overproduction results in cellular oxidative damage [73]. Plant components such as flavanol (quercetin) have shown anti-inflammatory and antioxidant properties and are promising candidates for colitis therapy [48,74,75]. In this regard, reduced ROS and H_2_O_2_ levels in colitic rats receiving QT-NPs indicated lowered lipid peroxidation and reduced free radical content. This was conceivably due to the fact that QT-NPs can depress the generation of ROS [32,76] by increasing cellular proficiency against oxidative stress with a consequent decline in lipid peroxidation and elevated numbers of healthy cells [77]. Accordingly, quercetin and resveratrol as naturally therapeutic molecules can hinder ROS, prevent the pro-oxidative damage, and cure oxidative stress-related diseases [78]. Moreover, quercetin can alleviate H_2_O_2_-induced cell damage, reduce intracellular ROS levels, and reverse the change in MDA levels [79]. Reduced plasma antioxidants and total intestinal antioxidant capacity [80], with higher MDA markers for lipid peroxidation [81], have been observed in colitic patients. Additionally, the higher levels of lipid peroxidation biomarkers observed after administration of DSS may imply a possible mechanism of tissue damage by reactive oxygen species [82]. Notably, the MDA content in colonic tissues declined with increasing levels of QT-NPs, signifying their protective role in lipid peroxidation.

Neutrophil infiltration activates overproduction of ROS and NO that ultimately provoke inflammation and intestinal injury [83]. Herein, it was noted that the destructive effects of ROS following administration of DSS were ameliorated by QT-NPs that prominently stimulated the antioxidant defense system. In this context, quercetin was demonstrated to attenuate T cell-mediated colitis in Rag1^−/^^−^ mice partially by modifying macrophage function by HO-1 [22]. Additionally, MPO activity, a good marker of leukocyte sequestration in inflamed colonic tissues as well as their activity, is linearly related to disease progression [84]. Remarkably, treatment of DSS-induced rats with higher levels of QT-NPs was effective in reducing MPO activity. Previously, administration of dietary quercetin or a combination comprising quercetin and quercetin monoglycoside restored reduced body weight and enhanced oxidative stress mediators including malonaldehyde myeloperoxidase and GSH in a 3% DSS-induced colitis model [85]. Moreover, treatment of the experimentally induced colitis models with active flavonoids greatly reduced neutrophil infiltration into the injured colonic tissue, as proved by a significant decline in colonic myeloperoxidase [86,87], thus contributing to the attenuation of intestinal inflammation. As crucial antioxidant enzymes, SOD, GPx and CAT comprise the key lines of cell defense against excess free radicals [14]. SOD is responsible for catalyzing O^2−^ into O_2_ and H_2_O_2_, while CAT and GPX can decompose H_2_O_2_ into O_2_ and H_2_O to remove excessive free radicals and ROS [88]. Quercetin is a potent antioxidant that secures the cells by altering endogenous antioxidant activities to alleviate apoptosis [74]. Taken together, flavonoids were able to reduce the oxidative stress in the experimental colitis models as evidenced by an improvement in different antioxidant markers together with decreased colonic lipid peroxidation or an augmentation of various enzyme activities with antioxidant functions [89]. The boosted role of QT-NPs using free quercetin in treating colitis, even with lower concentrations compared to previous studies, owed to their higher bioavailability and the protection from gastrointestinal conditions. Herein, loading of quercetin on chitosan nanoparticles enhanced its functions owing to the mucoadhesion, biocompatibility, permeation enhancement, and biodegradability of chitosan [90]. Moreover, there are many mechanisms that govern quercetin release from chitosan nanoparticles, such as diffusion of absorbed drugs, swelling of the polymer, and drug diffusion across the polymeric matrix [91]. Recently, attention was focused on the role of certain natural compounds that upregulate Nrf2, the nuclear factor erythroid 2-related factor 2-mediated antioxidant system, which is an important scavenger system that protects cells against oxidative stress and consequently ameliorates or prevents disorders [92]. Herein, the expression of HO-1 and Nrf2 was upregulated in the colitic group receiving QT-NPs, indicating their protection against oxidative stress, which agreed with Ju, Ge, Li, Tian, Wang, Zheng and Ju [22,93]. A previous report demonstrated that quercetin was potent in the augmentation of Nrf2-induced HO-1 protein expression in a murine model [93]. In this context, quercetin regulated redox signaling by upregulating the expression of Nrf2 and downregulating the expression of NFκB and cyclooxygenase-2 (COX-2) [13]. During colitis progression, pro-inflammatory cytokines are key biomarkers that are over-released in intestinal mucosa as a result of immune cell hyperactivation and are strongly linked to IBD pathogenesis. Excessive production of such inflammatory mediators promotes an inflammatory reaction that generates additional ROS and consequently augments more cytokine and proteolytic enzyme release, with subsequent tissue damage [94]. In the current study, the colitic non-treated group revealed an excessive level of pro-inflammatory and anti-inflammatory cytokines. In contrast, pro-inflammatory cytokines including IL-6, IL-1β, IFNγ and TNF-α were lowered, and anti-inflammatory cytokines (IL-10) were elevated in the group receiving QT-NPs, especially at higher levels, indicating their augmenting role in the amelioration of colonic epithelial damage. Accordingly, the ability of flavonoids to control the altered immune response after intestinal inflammation was proved by the remarkable decline in the increased levels of the various cytokines assessed in the inflamed colon [95,96]. The current results indicated excessive expression of IL-10 in the colon of rats with dextran sulfate-induced colitis, which was in agreement with [97]. Elevated IL-10 levels can be observed as a compensatory mechanism against colonic injury and are proposed to play a key role in limiting the inflammation of mucosa via lowering the production of MHC class II antigen and subsequent release of pro-inflammatory cytokines. Nevertheless, the higher IL-10 levels in the present study might not have been adequate to fully control the colon inflammation that accompanied the line of Autschbach et al. [98]. Moreover, the noted IL-10 restoration by quercetin-loaded NPs (closely similar to the control non-colitic group) suggested an enhancement of the inflammatory response that was associated with pro-inflammatory signal inhibition.

The mechanisms implicated in the promising effects of quercetin-loaded chitosan nanoparticles may be attributed to the properties of chitosan, such as its simplicity, non-toxicity, immune-stimulating activity, soft tissue compatibility, bio-adhesiveness, and low cost, as well as its insolubility in water at a higher pH [99]. Moreover, research has demonstrated that chitosan has mucoadhesive features and can strongly adhere to the mucus and thus expand its residence time mode of action in the GIT [100]. Additionally, chitosan is favored in nano formulation development to protect active cargo from unfavorable conditions in the GI tract during IBD treatment [101].

Both activated CD4^+^ and CD8^+^ T cells have been noted in the peripheral blood and intestinal mucosa of IBD patients during inflammation and are associated with other inflammatory markers [102,103]. Most mature T cells express either CD4 or CD8αβ glycoprotein, responsible for their division into two subpopulations, CD4^+^ or CD8^+^, respectively [104,105]. Moreover, the proportions of circulating CD4^+^ and CD8^+^ T lymphocytes are raised in patients with IBD compared with their proportions in controls [106]. Furthermore, increased plasma IL-6 and C-reactive protein levels together with higher levels of IL-2 and interferon-γ were detected with activated CD4^+^ and CD8^+^ T cells in colitic patients [106]. Herein, the most prominent downregulation of CD4 and CD8 gene expression was noticed in the colitic group treated with QT-NPs, demonstrating their efficacy in reducing excessive inflammatory reactions by suppressing the maturation of T lymphocytes. Similarly, the anti-inflammatory role of quercetin was considered to be linked to the development of CD4^+^ T cells in the murine colon [87]. Immune cells involved in inflammatory responses, such as monocytes [42], macrophages [43], and T cells [34], are modulated by quercetin through its inhibition of the pro-inflammatory transcription factors activating protein 1 (AP-1) and nuclear factor kB (NF-kB) [107]. Moreover, during the immune response, quercetin could inhibit CD4^+^ T-cell activation in IBD patients [22]. Herein, the superiority of QT-NPs in facilitating colonic epithelial amelioration by alleviating oxidative and inflammatory response could be complemented by improving its stability and long-lasting effects in gastrointestinal conditions. Patients suffering from IBD may have a dysfunctional epithelial barrier and increased tight junction permeability that enhance exposure to luminal antigens and microorganisms [108]. During experimental colitis, overproduction of ROS in mucosal cells can potentiate a cascade of inflammatory events that result in direct and indirect intestinal epithelial damage that disrupts the integrity of the intestinal mucosal barrier [109]. Several flavonoids, including quercetin, have been identified as protecting intestinal barrier function and modulating TJ regulation [110,111,112]. In the current work, the use of QT-NPs after induction of colitis played a significant role in the regulation of gene expression related to tight-junction proteins including occludin, *MUC-2* and *JAM*, unlike the DSS-induced colitic non-treated group, which exhibited downregulation of those genes. In accordance, overproduction of inflammatory cytokines as a consequence of IBD progression is among the main causes of the downregulation of tight junction-related genes [65,66]. Similarly, activation of TJ barrier function occurs following administration of quercetin owing to its antioxidant properties [113]. Quercetin supplementation may restore the expression of ZO-2, occludin, and claudin-1 and subsequently reduce the inflammation [114]. However, adequate amounts of quercetin are needed to attain its anti-inflammatory impact on the inflamed colon [111]. Therefore, the effective role of QT-NPs in augmenting the expression of tight junction-encoding genes could be related to their higher bioavailability when incorporated into nano form [92]. Additionally, significant histological damage following induction of IBD was detected in colons of the non-treated group as manifested by the complete destruction of colon epithelial architecture with diffuse inflammatory cell infiltration and loss of the colon crypts. Accordingly, typical histopathological changes were found in DSS-induced colitis comprising erosion/ulceration in epithelial tissues and infiltration of leukocytes into the lamina propria and submucosa with the depletion of mucin and goblet cells [115,116], signifying the excessive activation of the local immune responses. Additionally, the current histopathological alterations agreed with reports of DSS-induced colitis in a rat model of IBD [117]. The altered histoarchitecture of the colon after induction of colitis was prominently restored in the QT-NP-administered group, which agreed with [48], who found that quercetin can reduce intestinal oxidative damage and attenuate colitis.

Overproduction of ROS and cytokines occurs during colitis progression and has been found to trigger many transcription factors that promote the inflammatory response. Among them, nuclear factor kappa B (NFkB) triggers pro-inflammatory gene transcription involving *COX*-2 and *iNOS* [118]. COX-2 and iNOS are recognized as inducible enzymes that are rapidly expressed by fibroblasts and mononuclear macrophages and are closely associated with the colitis pathogenesis [119], as their excessive release can result in oxidative damage [120]. Flavonoids have the ability to attenuate inflammation by inhibition of cyclooxygenase-2 (COX-2) [121]. In the current study, the protein expressions of iNOS and COX-2 generated by DSS were markedly suppressed by treatment with QT-NPs. In this context, administration of phenolic compounds can suppress iNOS and COX2 expression [122,123]. As the activity of flavonoids depends on their absorption and bioavailability in the biological system [123], the incorporation of flavonoids into nanoparticles could enhance their specificity, stability, bioavailability, and biodistribution and subsequently augment their activities in alleviating IBD [124]. Herein, the marked downstream ability of inflammatory mediators such as *COX-2* and *iNOS* was regarded as an advantage of QT-NPs in colitis management. Moreover, these visible histological and immunohistochemical augmentations noted in the QT-NP group corroborated the biochemical, oxidative stress, intestinal integrity and gene expression findings reported in the current study and highlighted the prospective therapeutic potential of QT-NPs in decreasing the severity of IBD and reducing its related inflammatory response [125].

## 5. Conclusions

Alternative antioxidant therapy for colitis is considered to be a promising therapeutic candidate to prevent its severe progression. This is the first study to describe the protective impacts of QT-NPs on DSS-induced colitic rats. The most significant finding of the current study was that QT-NPs exerted potent benefits against the development of colitis. The beneficial actions of QT-NPs could be related to their incorporation in a nano-delivery system that protects them and promotes targeted delivery at the site of inflammation. Herein, the explored biochemical, immune and inflammatory markers revealed that QT-NPs could alleviate inflammation and oxidative stress mainly by controlling tight junction-related genes and redox imbalance and inflammation. Moreover, the positive role of QT-NPs, especially at higher concentrations, was prominent in modulating genes encoding Nrf2 and HO-1 and suppressing iNOS and COX-2 expression induced by DSS.

## Figures and Tables

**Table 1:** Scoring of Disease Activity Index (DAI).

	Body Mass Loss (%)	Stool Consistency	Rectal Bleeding
Scores	0 = None	0 = Normal consistency	0 = Negative
	1 = 0.1–5%		
	2 = 5–10%	2 = Loose stool	2 = Rectal occult blood
	3 = 10–20%		
	4 ≥ 20%	4 = Diarrhea	4 = Gross bleeding

**Table 2:** Primer sequences utilized for qRT-PCR analysis of targeted gene expression.

Target Gene	Primer Sequence (5′–3′)	Accession No./Reference
Occludin	F-CTGTCTATGCTCGTCATCG R-CATTCCCGATCTAATGACGC	NM-031329
*JAM*	F-GCTCAGCC ATACAGCAAATCC R-GGGAGTCGGGCAAT CATCAG	NM_017232
*MUC-2*	F-CAGAGTGCATCAGTGGCTGT R-CCCGTCGAAGGTGATGTAGT	XM_039101270.1
*Nrf-2*	F-GGTTGCCCACATTCCCAAAC R-GGCTGGGAATATCCAGGGCA	NM_031789.2
*HO-1*	F-CCCAGAGGCTGTGAACTCTG R-AGGCCCAAGAAAAGAGAGCC	NM_012580.2
*CD-4*	F-AGAAAGGACTGGCCAGAGAC R-CTGAAAGAGAAGCCTCGGCA	NM_031512.2
*CD-8*	F-ACTCACGGAGTGTGCTGAAG R-CCGCTCTGGCATCATCTTCA	NM_031539.2
*TLR-4*	F-TCCCACTCGAGGTAGGTGTT R-TTGTTAAGCTTATAAATCATGCGGCCTCAGG	NM_019178.2
β-actin	F-CGCAGTTGGTTGGAGCAAA R-ACAATCAAAGTCCTCAGCCACAT	V01217.1

Junction adhesion molecule (JAM), mucin-2 (MUC-2), Nuclear factor erythroid 2-related factor 2 (Nrf2), Heme oxygenase-1 (HO-1), gene encoding the CD4 membrane glycoprotein of T lymphocytes (CD-4), toll-like receptors (TLR4).

**Table 3:** Results of liver and kidney function and hematological tests in response to oral administration of different levels of quercetin nanoparticles (QT-NPs) in DSS-induced rat models of colitis.

Parameter	Control	DSS	DSS + QT-NPsI	DSS + QT-NPsII	DSS + QT-NPsIII	*p* Value	SEM
ALT (U/L)	37.12 ^c^	218.22 ^a^	135.80 ^b^	120.06 ^b^	60.30 ^c^	0.04	0.21
AST (U/L)	27.04 ^e^	193.23 ^a^	164.70 ^b^	98.73 ^c^	44.29 ^d^	0.02	0.31
Urea (U/L)	31.33 ^b^	48.58 ^a^	36.33 ^b^	32.76 ^b^	30.07 ^b^	0.01	0.19
Creatinine (U/L)	0.85 ^b^	1.25 ^a^	1.03 ^b^	1.04 ^b^	0.89 ^b^	<0.001	0.05
RBCs (×10^6^/μL)	13.23 ^a^	7.23 ^c^	7.63 ^c^	10.47 ^b^	11.37 ^b^	<0.001	0.10
Hb (g/dL)	12.16 ^a^	9.27 ^d^	10.57 ^c^	11.00 ^b^	11.95 ^ab^	<0.001	0.13

ALT: alanine transaminase, AST: aspartate transaminase, RBCs: red blood cells, Ht, hematocrit, Hb: hemoglobin. Mean values with different letters in the same row differ significantly at *p* < 0.05, SE: standard error. ^a–e^ Means of the rows with different letters were significantly different among groups (*p* < 0.05). Control: healthy non-colitic group: (rats were orally gavaged with PBS). Colitic groups including DSS (rats orally gavaged with dextran sodium sulphate), DSS + QT-NPsI (rats orally gavaged with DSS and quercetin nanoparticles (QT-NPs at the level 10 mg/kg body weight for 14 days)), DSS + QT-NPsII (rats orally gavaged with DSS and quercetin nanoparticles (QT-NPs at the level 15 mg/kg body weight for 14 days)), DSS + QT-NPsIII (rats orally gavaged with DSS and quercetin nanoparticles (QT-NPs at the level 20 mg/kg body weight for 14 days)). All groups were orally gavaged with 3% DSS.

## Data Availability

The data presented in this study are available on request from the corresponding author.
